# Supplementary material for: B-Cell Regeneration Profile and Minimal Residual Disease Status in Bone Marrow of Treated Multiple Myeloma Patients
Source: Cancers (Basel). 2021 Apr 3;13(7):1704. doi: 10.3390/cancers13071704 (PMC8038337; doi:10.3390/cancers13071704)
Supplement: Supplementary file 1 [file cancers-13-01704-s001.pdf]

Supplementary Materials:

B-Cell Regeneration Profile and Minimal Residual Disease Status in Bone Marrow of Treated Multiple Myeloma Patients

Robéria Mendonça de Pontes, Juan A. Flores-Montero, Luzalba Sanoja-Flores, Noemi Puig, Roberto J. Pessoa de Magalhães, Alba Corral-Mateos, Anna Beatriz Salgado, Omar García-Sánchez, José Pérez-Morán, Maria-Victoria Mateos, Leire Burgos, Bruno Paiva, Jeroen te Marvelde, Vincent H.J. van der Velden, Carlos Aguilar, Abelardo Bárez, Aranzazú García-Mateo, Jorge Labrador, Pilar Leoz, Carmen Aguilera-Sanz, Brian Durie, Jacques J.M. van Dongen, Angelo Maiolino, Elaine Sobral da Costa and Alberto Orfao on behalf of the EuroFlow Consortium

Table S1. Clinical and laboratory features observed at diagnosis for the MM patients included in this study (n = 162).

Disease Features		% Patient Distribution
**Sex**	Male	57%
	Female	43%
**Age at diagnosis**	< 65y	62%
	≥ 65y	38%
**ISS stage**	I	39%
	II	30%
	III	31%
**Cytogenetic risk***	High	29%
	Low	71%
**Serum β2-microglobulin (mg/L)**	<5.5	69%
	≥ 5.5	31%
**Albumin (mg/L)**	< 3.5	34%
	≥ 3.5	66%
**LDH (U/L)**	Normal	65%
	Increased	35%
**Type of M-component**	IgG	61%
	IgA	19%
	IgD	1%
	BJ	13%
	Oligosecretory	3%
	Non-secretory	3%

Abbreviations: ISS, international staging system; LDH, lactate dehydrogenase; BJ, Bence-Jones; NA, not available. High-risk cytogenetic include del(17/17p), t(4;14), t(14;16) and/or, t(14;20). *not available in 59/162 cases.

Table S2. Distribution of maturation-associated normal/residual B-cell and nPC cell population in BM of HD *vs*. MM patients studied at diagnosis and at different time points after therapy.

Cell population (%)	HD (n = 14)	Multiple myeloma At diagnosis (n = 25)	Multiple myeloma End of Induction (n = 27)	Multiple myeloma Post-ASCT (day + 100) (n = 94)
**Total B-cells**	**2.6** (1–4.6)	**1.2** [a] (0.4–3.3)	**0.8** [a] (0.3–5)	**3.3** [b] (0.05–11.4)
**Pre-germinal center B-cells**	**1.7** (0.4–3.3)	**0.5** [a] (0.07–2.2)	**0.5** [a] (0.1–4.5)	**3.1** [a, b] (0.003–11.2)
BCP	**0.3** (0.09–1.7)	**0.01** [a] (<0.0002–0.8)	**0.3** [b] (<0.0002–2.3)	**1.5** [a, b] (0.002–10.3)
Stage I BCP	**0.02** (0.006–0.2)	**0.0005** [a] (<0.0002–0.1)	**0.02** [b] (<0.0002–0.7)	**0.2** [a, b] (<0.0002–0.9)
Stage II BCP	**0.3** (0.09–1.5)	**0.01** [a] (<0.0002–0.7)	**0.2** [b] (<0.0002–2.1)	**1.3** [a, b] (0.002–9.4)
Stage I / stage II BCP ratio	**0.1** (0.04–0.2)	**0.02** [a] (0–0.2)	**0.1** [b] (0 –1.6)	**0.09** [b] (0 –1.2)
Transitional/naive B-cells	**1.1** (0.3–2)	**0.4** [a] (0.05–1.9)	**0.2** [a, b] (0.004–2.5)	**1.5** [b] (0.0008–5.7)
**Post-germinal center B-cells**	**0.8** 0.4–1.4)	**0.4** [a] (0.1–1.6)	**0.2** [a, b] (0.03–0.6)	**0.1** [a, b] (0.009–0.9)
Memory B-cells	**0.4** (0.06–1.3)	**0.3** (0.05–1.5)	**0.07** [a, b] (0.005–0.5)	**0.03** [a, b] (0.0005–0.5)
nPC	**0.3** (0.08–0.9)	**0.04** [a] (0.005–0.5)	**0.06** [a] (0.008–0.4)	**0.08** [a, b] (0.002–0.8)
$CD19^+$ nPC	**0.2** (0.03–0.6)	**0.03** [a] (0.002–0.4)	**0.05** [a] (0.006–0.4)	**0.07** [a, b] (<0.0002–0.8)
$CD19^-$ nPC	**0.1** (0.02–0.3)	**0.006** [a] (<0.0002–0.08)	**0.009** [a] (<0,0002–0.07)	**0.007** [a] (<0.0002–0.2)
$CD19^+$/$CD19^-$ nPC ratio	**2** (0.7–8.6)	**2.2** (0–18.6)	**5.8** [a, b] (0 –147)	**8.6** [a, b] (0 – 42.4)
**Mature B-cells ¥**	**1.5** (0.4–3)	**0.8** [a] (0.1–2.8)	**0.4** [a, b] (0.009–2.6)	**1.5** [b] (0.004–5.9)
**BCP/Mature B-cell ratio**	**0.2** (0.08–1.6)	**0.005** [a] (0–2.5)	**0.6** [b] (0–30.2)	**1.1** [a, b] (0–13.2)

Abbreviations: BCP, B-cell precursors; nPC, normal plasma cell; ¥ Mature B-cells (transitional/naïve B-cells plus memory B-cells); limit of detection of MRD of 0.0002% (2×10^{-6}); a *p*-value < 0.05 *vs*. HD, b *p* < 0.05 *vs*. MM at diagnosis (Mann Whitney-U test).

Table S3. Distribution of maturation-associated normal/residual B-cell and nPC cell populations in BM of MM patients studied at diagnosis and at different time points after therapy according to the patients' cytogenetic risk (standard *vs*. high risk).

Cell population (%)	At diagnosis (n = 21)		End of Induction (n = 22)		Post ASCT (day + 100) (n = 45)	
	Standard risk (n = 18)	High risk (n = 3)	Standard risk (n = 12)	High risk (n = 10)	Standard risk (n = 35)	High risk (n = 10)
**Total B-cells**	**1** (0.4–2.1)	**1.4** (0.5–3.3)	**0.7** (0.25–2.7)	**0.7** (0.3–5)	**2.5** (0.6–11.4)	**2.7** (1.1–4.4)
**Pre-germinal center B-cells**	**0.4** (0.07–1.7)	**1.1** (0.1–2.2)	**0.4** (0.1 -2.7)	**0.3** (0.1–4.5)	**2.4** (0.5–11.2)	**2.6** (1.07–4.2)
BCP	**0.009** (<0.0002–0.4)	**0.4** (<0.0002–0.8)	**0.2** (0.01 -2.3)	**0.07** (<0.0002 -2.2)	**1.2** (0.2–10.3)	**1.4** (0.7–2.6)
Stage I BCP	**0.0003** (<0.0002–0.06)	**0.02** (<0.0002–0.1)	**0.02** (0.001–0.3)	**0.02** (<0.0002–0.7)	**0.2** (0.009–0.9)	**0.1** (0.04–0.4)
Stage II BCP	**0.008** (<0.0002-0.4)	**0.3** (<0.0002–0.7)	**0.2** (0.01 -2.1)	**0.05** (<0.0002–2)	**1** (0.1–9.4)	**1.2** (0.5–2.3)
Stage I/stage II BCP ratio	**0.01** (0–0.2)	**0.07** (0–0.2)	**0.1** (0.02–0.2)	**0.3** (0– 0.8)	**0.1** (0.02–1.2)	**0.1** (0.02–0.6)
Transitional/naive B-cells	**0.4** (0.05–1.4)	**0.3** (0.1–1.9)	**0.2** (0.02–0.6)	**0.2** (0.004–2.5)	**1.3** (0.2–4.9)	**1.1** (0.3–2.1)
**Post-germinal center B-cells**	**0.4** (0.09–1.2)	**0.4** (0.2–1.1)	**0.2** (0.08–0.6)	**0.2** (0.03–0.6)	**0.08** (0.02 -0.5)	**0.08** (0.03 -0.2)
Memory B-cells	**0.07** (0.007–0.3)	**0.09** (0.005–0.5)	**0.07** (0.007–0.3)	**0.09** (0.005–0.5)	**0.03** (0.006–0.5)	**0.03** (0.008–0.1)
nPC	**0.03** (0.005–0.5)	**0.1** (0.01–0.1)	**0.06** (0.02–0.4)	**0.1** (0.008 -0.4)	**0.05** (0.002–0.2)	**0.04** (0.01–0.2)
$CD19^{+}$ nPC	**0.03** (0.002–0.4)	**0.07** (0.005–0.1)	**0.05** (0.02–0.4)	**0.07** (0.007–0.4)	**0.04** (0.0008–0.2)	**0.03** (0.01–0.2)
$CD19^{-}$ nPC	0.005 (<0.0002–0.008)	**0.05** (0.007–0.05)	**0.009** (0.002 -0.06)	**0.003** (0.0006 -0.04)	**0.005** (0.003–0.06)	**0.007** (0.02–0.05)
$CD19^{+}$/$CD19^{-}$ nPC ratio	**4.5** (0–18.6)	**1.4** (0.7–2.1)	**6.9** (2.9–19.3)	**7.2** (2.7–147)	**6.9** (0–29.2)	**8.3** (3.7–19.4)
**Mature B-cells ¥**	**0.8** (0.1–1.8)	**0.6** (0.3–2.8)	**0.3** (0.03–0.7)	**0.5** (0.009–2.6)	**1.4** (0.3–5)	**1.2** (0.3–2.2)
**BCP/Mature B-cell ratio**	**0.004** (0–2.5)	**0.1** (0–1.4)	**0.9** (0.03–5.9)	**0.2** (0–30.2)	**0.9** (0.1–10.4)	**1.5** (0.4–3.2)

Abbreviations: BCP, B-cell precursors; nPC, normal plasma cell; ¥ Mature B-cells (transitional/naïve B-cells plus memory B-cells); limit of detection of MRD of 0.0002% (2×10^{-6}); *, $p < 0.05$ for comparison between MM samples standard risk *vs*. high-risk (Mann Whitney-U test). High-risk cytogenetic include del(17/17p), t(4;14), t(14;16) and/or, t(14;20).

Table S4. Distribution of maturation-associated normal/residual B-cell and nPC cell populations in BM of MM patients studied at day+100 post-autologous stem cell transplantation, according to the type of induction therapy received.

Cell Population (%)	Post ASCT (day+100) (n=93)	
	**PI plus IMIDs plus steroids (n = 43)[a]**	**PI plus steroids or IMIDs plus steroids (n = 50)[b]**
**Total B-cells**	**2.5** (1.1–11.4)	**4.3 *** (0.05–11)
**Pre-germinal center B-cells**	**2.4** (0.5–11.2)	**4 *** (0.003–11)
BCP	**1.2** (0.06–12.3)	**1.9 *** (0.002 -6.2)
Stage I BCP	**0.2** (0.002–0.9)	**0.2** (<0.0002 -0.6)
Stage II BCP	**1** (0.06–9.4)	**1.8 *** (0.002–6)
Stage I/stage II BCP ratio	**0.1** (0.02 -1.2)	**0.8 *** (<0.0002–0.5)
Transitional/naive B-cells	**1.3** (0.09–5)	**1.7 *** (0.0008–6)
**Post-germinal center B-cells**	**0.1** (0.02 -0.6)	**0.2 *** (0.009–0.9)
Memory B-cells	**0.03** (0.005–0.5)	**0.03** (0.005–0.2)
nPC	**0.05** (0.02–0.6)	**0.1 *** (0.008–0.8)
$CD19^+$ nPC	**0.04** (0.008–0.4)	**0.1 *** (<0.0002–0.8)
$CD19^-$ nPC	**0.007** (0.003–0.2)	**0.01** (<0.0002 -0.1)
$CD19^+/CD19^-$ nPC ratio	**6.3** (<0.0002–24.1)	**11** (<0.0002–42.4)
**Mature B-cells ¥**	**1.3** (0.1–5)	**1.8 *** (0.004–6)
**BCP/Mature B-cell ratio**	**0.9** (0.09–13.2)	**1.2** (<0.0002–4)
**% (n/n) MRD+ status**	60% (26/43)	58% (29/50)
**PFS in months**	32	NR

Abbreviations: BCP, B-cell precursors; nPC, normal plasma cell, MRD minimal residual disease, PFS progression free-survival; ¥ Mature B-cells (transitional/naïve B-cells plus memory B-cells); limit of detection of MRD of 0.0002% (2x10-6); * $p < 0.05$ for comparison between different of types of protocols induction to prior ASCT (Mann Whitney-U test). [a] in this group was included: PI+IMIDs+steroids (n = 40), PI+IMIDs+steroid+MoAb (n = 1), PI+IMIDs+steroids+Chemotherapy (VDL-Page, n = 2); [b] in this group was included: PI+ cyclophosphamide+steroid (n = 22), cyclophosphamide+IMIDs+steroirds (n = 19), PI+IMIDs+steroids+ cyclophosphamide (n = 3) PI+steroid (n = 3), IMID+steroid (n = 2).
